# High Shear Stress‐Induced Endothelial Piezo1 Downregulation Promotes Intracranial Aneurysm Formation via the PDGF‐BB/PDGFRβ Paracrine Signaling Pathway

**DOI:** 10.1002/cns.70715

**Published:** 2025-12-28

**Authors:** Zhiwen Lu, Sisi Li, Fengfeng Xu, Haishuang Tang, Shijie Zhu, Chuanchuan Wang, Xiaohua Yang, Qinghai Huang

**Affiliations:** ^1^ Department of Neurosurgery, Naval Medical Center The PLA Naval Medical University Shanghai China; ^2^ Department of Neurovascular Intervention, Clinical Center of Neuroscience, Shanghai General Hospital Shanghai Jiao Tong University School of Medicine Shanghai China; ^3^ Department of Neurosurgery, Tiantan Hospital Capital Medical University Beijing China; ^4^ Department of Neurovascular Centre, Changhai Hospital Naval Medical University Shanghai China; ^5^ Central Laboratory, Shanghai Chest Hospital Shanghai Jiao Tong University School of Medicine Shanghai China; ^6^ Department of Neurosurgery, Huashan Hospital Fudan University Shanghai China

**Keywords:** intracranial aneurysm, phenotype transformation, Piezo1, platelet‐derived growth factor subunit BB (PDGF‐BB), shear stress

## Abstract

**Background:**

Abnormally high shear stress (HSS) is strongly associated with intracranial aneurysm (IA) formation. Endothelial Piezo1 is sensitive to shear stress stimulation, but the mechanism by which it mediates this mechanobiological coupling process is unclear.

**Methods:**

The correlation between shear stress and the Piezo1 expression was investigated using human IA samples and a parallel‐plate flow chamber system. To determine the effects of endothelial Piezo1 on the phenotype of neighboring vascular smooth muscle cells (VSMCs) and IA formation, the CRISPR/Cas9 system was used to inhibit endothelial Piezo1 gene expression in vitro. Piezo1^ΔEC^ mice were produced by injecting AAV2‐BR1‐Tie2‐Cre into 8‐week‐old male Piezo1^flox/flox^ mice, which were further used to construct the IA mouse model. Single‐cell RNA sequencing and intercellular communication analyses of co‐cultured endothelial cells (ECs) and VSMCs were used to screen for receptor‐ligand pairs after inhibiting EC Piezo1 in vitro. The role of the screened receptor‐ligand pair was further validated via in vivo and in vitro experiments. Additionally, the underlying mechanisms were investigated.

**Result:**

Piezo1 expression correlated negatively with the shear stress in human IA. HSS reduced EC Piezo1 expression and promoted VSMC phenotypic transformation compared with physiological shear stress. Depletion of EC Piezo1 resulted in the VSMC phenotypic transformation and, more importantly, promoted aneurysmal vascular remodeling in the mouse IA model. The platelet‐derived growth factor subunit B (PDGFB)_Platelet‐derived growth factor receptor β (PDGFRβ) was identified as being involved in this process. Moreover, the PDGFRβ antagonist reversed the VSMC phenotypic transformation and attenuated IA progression. Mechanistically, Piezo1 depletion promoted PDGFB expression via YAP/β‐catenin pathway.

**Conclusion:**

HSS downregulates Piezo1 expression in ECs, which subsequently enhances PDGF‐BB expression through the YAP/β‐catenin signaling pathway. The elevated PDGF‐BB facilitates phenotypic transition of VSMCs via PDGFRβ binding, ultimately contributing to IA formation.

## Graphical abstract

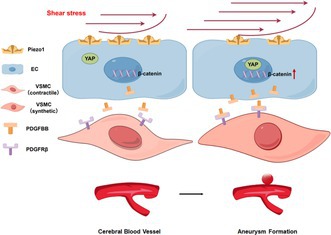

**Abbreviations**
CFD: computational fluid dynamics
DEGs: differentially expressed genes
DEME: Dulbecco's Modified Eagle Medium
EC: endothelial cell
ECM: extracellular matrix
ELISA: enzyme‐linked immunosorbent assay
FBS: fetal bovine serum
GO: gene ontology
HE: hematoxylin–eosin
HSS: high shear stress
IA: intracranial aneurysms
IEL: internal elastic lamina
IHC: immunohistochemistry
KEGG: Kyoto encyclopedia of genes and genomes
MMP2: matrix metallopeptidase 2
OPN: Osteopontin
PDGFB: platelet‐derived growth factor subunit B
PDGFRβ: platelet‐derived growth factor receptor β
PSS: physiological shear stress
SM22α: smooth muscle 22 alpha
TGF‐β: transforming growth factor‐β
VSMC: vascular smooth muscle cell
WB: western blotting
α‐SMA: alpha‐smooth muscle actin

## 1 Background

Intracranial aneurysm (IA) is characterized by cystic pathological dilatation of intracranial arteries. Subarachnoid hemorrhage is a devastating consequence of IA rupture and is associated with high mortality and disability rates [1]. The precise etiology of IA remains unknown. However, it is widely accepted that genetic and environmental factors, particularly abnormal hemodynamics, promote the development of IA [2, 3, 4]. During this process, vascular smooth muscle cells (VSMCs) transform from contractile to synthetic type following the internal elastic lamina (IEL) disruption to synthesize collagen and extracellular matrix (ECM). Although the remodeling of collagen fibers increases the strength of the vessel wall to counteract the increasing mechanical stress, it also causes localized bulging of the arterial wall, known as aneurysmal wall remodeling [5].

Hemodynamics, represented by shear stress, plays an important role in the natural history of IA [6, 7, 8]. Physiological levels of shear stress contribute to the normal function of blood vessels [9, 10]. However, responding to a chronic high shear stress (HSS) stimulation, an adaptive dilatational remodeling of the vessel wall occurs to maintain the shear stress baseline level [11, 12]. At this point, vascular remodeling is dominated by an increased lumen diameter with relatively little change in wall thickness and is accompanied by micro‐disconnections of the IEL and VSMC proliferation [13]. Similar phenomena have also been observed in the initial IA [14, 15]. However, the mechanism underlying HSS‐induced pathological remodeling of IA vessels remains unclear.

Piezo1, a novel mechanosensitive ion channel, is widely distributed in blood vessels [16]. Piezo1 converts the shear stress stimulus of blood flow into electrical and chemical signals via Ca^2+^ influx and regulates several physiological and pathological processes [17, 18, 19, 20]. Specific disruption of Piezo1 in endothelial cells (ECs) during embryonic development retards embryonic growth and inhibits the normal yolk sac vasculature development without stopping the heartbeat, suggesting that Piezo1 contributes to vascular maturation and differentiation via shear stress [17]. In mature vessels, EC Piezo1 senses turbulence and activates integrins via P2Y2 receptors and Gq/11, subsequently activating pro‐atherosclerotic signaling via focal adhesion kinase and nuclear factor kappa‐B [20]. This ultimately leads to intimal hyperplasia based on a pathological basis of VSMC proliferative migration and increased ECM secretion. Furthermore, low osmolarity‐mediated stretching of EC membranes upregulates Jagged1 expression via the Piezo1/Ca^2+^/AKT/mTOR signaling pathway in the pulmonary artery [21]. The binding of Jagged1 ligands to Notch3 receptors in neighboring VSMCs further modulates the VSMC phenotype, which may be related to vascular remodeling in pulmonary hypertension [22, 23]. These results suggest that EC Piezo1 is involved in vascular remodeling by regulating the VSMC phenotype. However, direct experimental evidence for this assumption is lacking.

In this study, we hypothesized that EC Piezo1‐mediated HSS stimulation results in VSMC phenotypic transformation and subsequently promotes IA formation through intercellular communication.

## 2 Materials and Methods

### 2.1 Patients and Samples

The Ethical Committee of Naval Medical Center approved this study (No. 2025Q050). Informed consent was obtained from the patients. A total of 13 patients with IAs were enrolled in this study (Table S1). IA specimens were obtained during surgery. Tissue samples were collected using RNAlater (Thermo Fisher Scientific, USA) and stored at −80°C for high‐throughput sequencing.

### 2.2 Computational Fluid Dynamics Analysis

The patient‐specific cerebrovascular model was reconstructed before surgery using Siemens (Germany) or Philips (Netherlands) imaging machines and introduced into the GeomagicStudio 2012 (Geomagic, USA). The aneurysms and parent arteries were then smoothed and meshed. ANSYS ICEM computational fluid dynamics (CFD)14.0 (ANSYS, USA) software was used to separate the aneurysm from the parent artery, set boundary conditions, and perform calculations. The shear stress was determined using MATLAB 7.0 and ANSYS CFX 14.0 software packages. To standardized the shear stress in different patients, the ratio of the shear stress of the aneurysm and parent artery was used (Table S2).

### 2.3 Cells and Cell Culture

Murine endothelial cells (ECs) and vascular smooth muscle cells (VSMCs) were isolated from the aortas of healthy C57BL/6 mice, following previous method [24]. Briefly, the aortas were meticulously excised and freed from surrounding connective tissues. Then the aortas were digested for 30 min at 37°C in Dulbecco's Modified Eagle Medium (DMEM) supplemented with 200 U/mL type III collagenase, 0.1 mg/mL elastase, and 0.5 mg/mL soybean trypsin inhibitor. Thirdly, they were incubated by an additional 45 min incubation at 37°C in a medium containing 130 U/mL type III collagenase, 0.1 mg/mL elastase, and 0.5 mg/mL soybean trypsin inhibitor. The connective tissue fragments were further cut into small pieces and digested at 37°C for another 60 min. VSMC were subsequently harvested via centrifugation at 200 × *g* for 3 min and were grown in DMEM enriched with 20% fetal bovine serum (FBS), 100 U/mL penicillin, and 100 U/mL streptomycin. To extract ECs, the aorta was longitudinally opened and sectioned into small pieces. These segments were placed on a fibronectin‐coated culture dish with intima facing down and cultured in M199 medium containing 20% FBS, 25 U/mL heparin, 10 ng/mL ECGF, 100 U/mL penicillin, and 100 U/mL streptomycin at 37°C in 5% CO_2_. ECs began to migrate from the aortic explants after about five days. Both ECs and VSMCs with a cytological age of 5–8 were used in the study.

### 2.4 Parallel‐Plate Flow Chamber System

Endothelial cell was seeded on the outer surface of a 10‐μm‐thick porous membrane coated with rat‐tail collagen type I (C8062, Solarbio, Beijing, China). VSMC was co‐cultured with endothelial cell for 24 h on the inner surface of the membrane. The two cell types were incorporated into a parallel‐plate flow chamber for 24 h as previously reported. Shear stress was applied to endothelial cell layer [25].

### 2.5 HSS Construction

**FIGURE 1:**
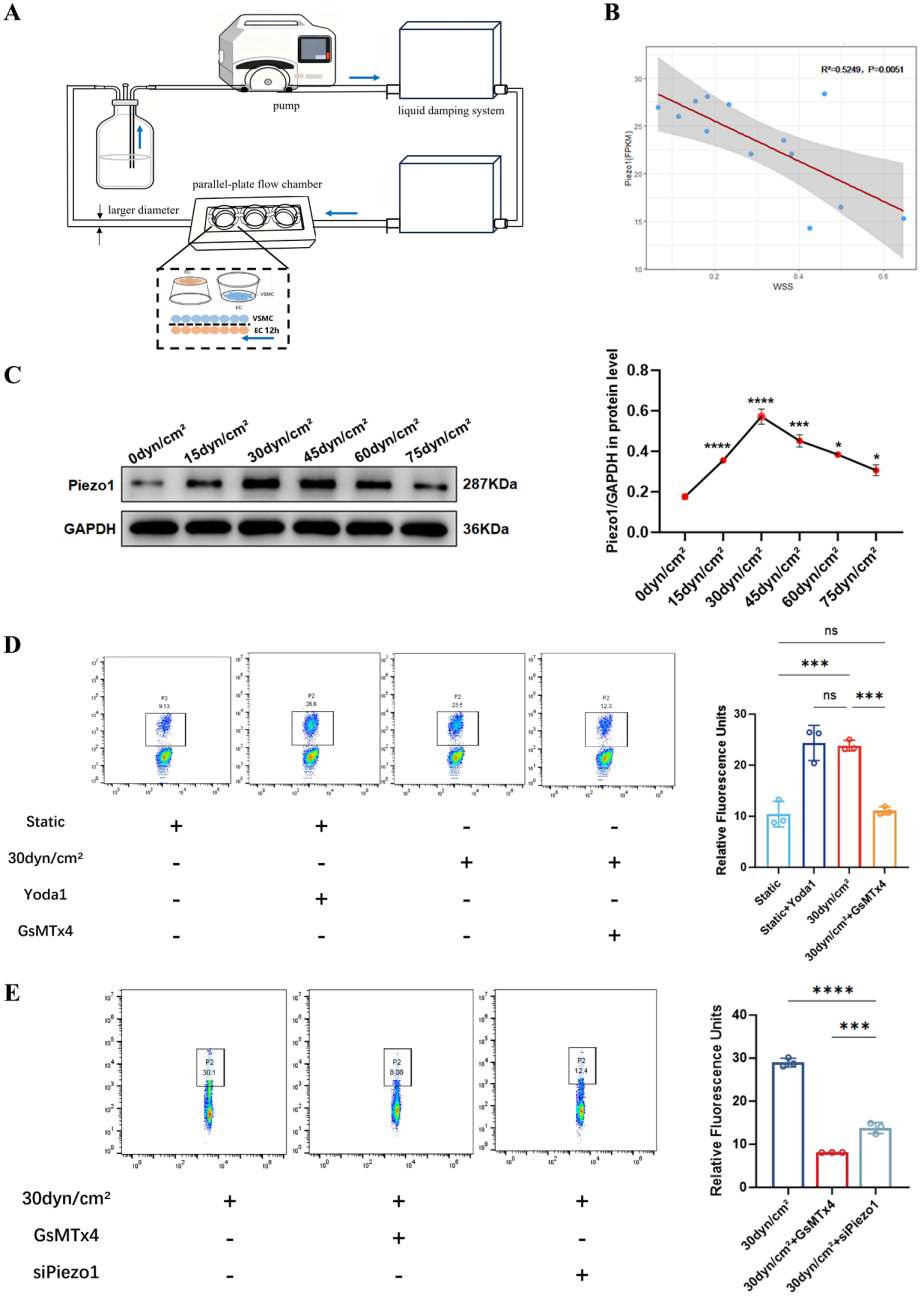
Shear stress regulates Piezo1 expression and activates Piezo1 ion channel. (A) The schematic illustrates the construction of a HSS stimulation model. (B) Correlation between Piezo1 gene expression and normalized wall shear stress in human IA tissue (*n* = 13). (C) The protein levels of Piezo1 in ECs at different shear stress stimulation. (D) Fluorescence intensity of free calcium ions in ECs under various stimulus conditions. (E) Effect of the Piezo1 channel inhibitor (GsMTx4) and disrupting Piezo1 gene on calcium ion fluorescence intensity in shear stress‐stimulated ECs. *, ***, and **** represent *p*‐values < 0.05, 0.001, and 0.0001 respectively.

HSS was achieved by improving the parallel‐plate flow chamber system. Specifically, the tube connecting the flow chamber units was replaced with one having a larger diameter to provide a higher flow rate. Two liquid damping systems were connected in series to reduce the oscillatory shear index. Then, the time of shear stress stimulation was reduced to 12 h to avoid EC loss during high‐flow flushing. The schematic is shown in Figure 1A.

### 2.6 Establishment of Piezo1 Knockout in Primary Endothelial Cells

The Piezo1 gene deleted using the CRISPR/Cas9 system. According to the mouse Piezo1 (NM_001357349) sequence, three candidate sgRNAs targeting Piezo1 were synthesized using Genechem (GenePharma Co Ltd., Shanghai, China). The sequences of sgRNAs are reported in Table S3. Lentivirus (Lenti‐Cas9‐sgRNA) encoding Cas9 nuclease and guide RNA targeting Piezo1 or the vector for wild‐type control were constructed and packed using Genechem (Shanghai, China). Endothelial cells were transfected with Lenti‐sgRNA‐Cas9 for three days and then selected using Puromycin to generate a polyclonal Piezo1 −/− cell line (EC^siPiezo1^).

### 2.7 Measurement of Intracellular Ca^2+^

Intracellular Ca^2+^ flux was detected using a Fluo‐4‐AM staining kit (S1060, Beyotime, China) and measured using flow cytometry or a confocal microscope. ECs were first co‐cultured with VSMCs as previously described and then labeled with 5 μM Fluo‐4‐AM in a 6 well‐plate. After incubation at 37°C protected from light for 1 h, ECs were washed once with PBS. The cells were digested and then centrifuged at 500 *g* for 5 min at room temperature after 1 h of shear stress stimulation. The cells were resuspended by adding 0.5 mL assay buffer. The intracellular Ca^2+^ level was measured at an excitation wavelength of 488 nm and an emission wavelength of 530 nm using a flow cytometer. Additionally, to determine the Ca^2+^ concentrations in ECs following BAPTA‐AM and CaCl_2_ interventions, the mono‐cultured ECs incubated with Fluo‐4‐AM were monitored using confocal microscopy. Image Pro Plus software 6.0 (Medical Cybernetics, USA) was used to measure the mean density (MD = integrated optical density/area).

### 2.8 Intervention With Yoda1, GsMTx4, BAPTA‐AM, CaCl_2_ , and Imatinib Mesylate

Yoda1 (Piezo1 agonist), GsMTx4 (Piezo1 antagonist), BAPTA‐AM (an intracellular calcium chelator), and CaCl_2_ were all used to treat ECs before shear stress stimulation. Specifically, 0.2 μM of Yoda1 (Abcam, USA) or 2.5 μM of GsMTx4 (Abcam, USA) were applied to the EC layer immediately after Fluo‐4‐AM incubation [17]. These cells were either left in a resting state or stimulated by shear stress for 30 min before calcium ion concentrations were measured. To detect VSMC phenotypes, ECs were treated with the same doses of Yoda1 followed by co‐culture in a resting state for 1 h. CaCl_2_ (1.5 mM, Sinopharm, China) and BAPTA‐AM (10 μM, MCE, USA) were added to the culture medium of ECs before shear stress stimulation to alter the intracellular free Ca^2+^ concentration [26]. Imatinib mesylate (5 μM, Selleck, USA) was applied to VSMCs for 90 min before treatment with EC medium to elucidate the receptor‐ligand pair between ECs and VSMCs [27]. To further investigate the effects of the receptor‐ligand pair on IA, Piezo1^ΔEC^ mice were administered 50 mg/kg [28] imatinib (Cat. No. #HY‐15463, MCE, USA) in 0.5% DMSO intraperitoneally daily for three weeks after stereotactical brain injection.

### 2.9 Generation of Conditional EC Piezo1 Knock‐Out Mice

Two pairs of C57BL/6 Piezo1^flox/flox^ mice were purchased from the Research Center of the Southern Model Organisms (Shanghai, China) and bred to produce homozygous offspring. Eight‐week‐old male mice were used to generate conditional EC Piezo1 knock‐out mice (Piezo1^ΔEC^) by injecting of AAV2‐BR1‐Tie2‐Cre (1.308 × 10^12^v.g.) in 200 μL PBS via the tail vein (*n* = 24). The negative controls were administered AAV2‐BR1‐Tie2 at the same dose (*n* = 18). AAV2‐BR1‐Tie2‐Cre and AAV2‐BR1‐Tie2 were constructed and packaged by Genechem Company (Shanghai, China). The construction of the IA model was performed four weeks after Adeno‐associated virus (AAV) injection.

### 2.10 Immunofluorescence

Double‐immunofluorescence staining was performed as previously described [29]. Mice were intracardially perfused with PBS. Brain samples were extracted and fixed for 24 h with 4% paraformaldehyde, and then dehydrated with a 30% sucrose solution. Frozen brain samples were cut into coronal sections (10‐μm thick) using a cryostat (CM3050S; Leica Microsystems, Germany). After washing thrice with PBS for 10 min each, brain sections were incubated with 5% donkey serum for 2 h at room temperature and then incubated overnight at 4°C with the following primary antibodies: anti‐Piezo1 antibody (1:200, rabbit, 15939‐1‐AP, Proteintech, USA), anti‐CD31 antibody (1:100, rat, 65058‐1‐Ig, Proteintech, USA), anti‐α‐SMA antibody (1:500, rabbit, ab124964, Abcam, USA), anti‐SM22α antibody (1:50, rabbit, 10493‐1‐AP, Proteintech, USA), anti‐OPN antibody (1:50, rabbit, 22952‐1‐AP, Proteintech, USA), and anti‐MMP2 antibody (1:400, rabbit, 10373‐2‐AP, Proteintech, USA). One day later, the sections were incubated with fluorescent‐conjugated goat anti‐rabbit IgG (1:500, A0562, Beyotime, China) or goat anti‐rat IgG (1:500, A0192, Beyotime, China) for 1 h at room temperature. The nuclei were stained with 4′,6‐diamidino‐2‐phenylindole (C1006, Beyotime, China) following the manufacturer's instructions. Confocal images were taken using a fluorescence microscope (Leica Microsystems, Germany).

### 2.11 IA Mouse Model

All animal experiments were conducted according to the guidelines of the Directive 2010/63/EU of the European Parliament on the protection of animals used for scientific purposes and approved by the Ethical Committee of the Naval Medical Center (No. 2025Q050). Twelve‐week‐old male control and Piezo1^ΔEC^ mice were subjected to IA modeling and categorized as the control and Piezo1^ΔEC^ groups, respectively. Additionally, same‐aged C57BL/6 male mice (*n* = 18) were used as a sham group. Mice were anesthetized before surgery by intraperitoneal injection of pentobarbital sodium (50 mg/kg, Butler). The IA model was constructed according to our previously reported methods [30]. Briefly, the left common carotid and left renal arteries were ligated. One week later, 35 mU of elastase (Sigma, USA) was stereotactically injected into the right chiasmatic cistern (0.7 mm to the right of the fontanel point, 1.2 mm anteriorly, and 6.2 mm deep) one week later. After three days of injection, the mice were fed a hypertension‐inducing diet. They were then perfused with PBS and bromophenol blue gelatin through the heart under deep anesthesia three weeks later. In the end, the mice were euthanized in a CO_2_ chamber. To evaluate the severity of aneurysmal vascular remodeling, the vessels were classified into four classes according to the previous study [31]. An aneurysm was defined as an outpouching of weakened vessel walls at the circle of Willis, with a diameter 150% greater than that of the normal artery [32]. In addition, mouse magnetic resonance angiography was used to assess the effects of imatinib on IA in Piezo1^ΔEC^ mice.

### 2.12 Hematoxylin–Eosin (HE) Staining and Immunohistochemistry (IHC)

Mouse brain tissue samples were embedded in paraffin and cut into sections, 5 μm thick. HE and IHC staining was performed as previously described [33, 34]. The primary antibodies used were as follows: α‐SMA (ab12496, Abcam, USA), SM22α (10493‐1‐AP, Proteintech, USA), OPN (22952‐1‐AP, Proteintech, USA), and MMP2 (10373‐2‐AP, Proteintech, USA). Following the manufacturer's instructions, 3,3′‐diaminobenzidine (DAB) plus chromogen (Thermo Fisher Scientific, MA) was used for substrate visualization. All slides were observed at 400× magnification. MD was measured using Image‐Pro‐Plus software 6.0 (Medical Cybernetics, USA).

### 2.13 Single‐Cell RNA Sequencing

EC^siPiezo1^ and EC^siControl^ were co‐cultured with VSMC under HSS stimulation for 12 h. Cells were then isolated and added to a mixture of fresh cell lysate (1 μL, Genefund Biotech Co. Ltd., Shanghai) and DPEC water for single‐cell RNA‐seq. Full‐length single‐cell RNA‐seq libraries were prepared using the Smart‐seq2 protocol [35, 36]. The library was then subjected to Illumina sequencing with paired‐end 2 × 150 as the sequencing mode by Genefund (Shanghai, China). Raw reads were filtered to obtain high‐quality clean reads by removing sequencing adapters, short reads (length < 35 bp), and low‐quality reads using Cutadapt v1.18 and Trimmomatic v0.35 [37]. FastQC was then used to ensure high read quality [38]. The clean reads were mapped to the mouse genome using the HISAT2 v2.1.0 software [39]. Gene expression levels were estimated using fragments per kilobase of exon per million fragments (FPKM) mapped using StringTie v1.3.4d [40]. Differential gene expression was measured using edgeR v3.24.2, an R package [41]. The false discovery rate control method was used to calculate the adjusted *p*‐values in multiple tests to evaluate the significance of differences. Only genes with an adjusted *p*‐value < 0.05 and |log2FC| ≥ 1 were used for subsequent analysis. The gene annotation file was retrieved from the Ensembl genome browser 96 database (http://www.ensembl.org/index.html). ClusterProfiler v3.4.4 (R package) was used to annotate genes with gene ontology (GO) terms and Kyoto Encyclopedia of Genes and Genomes (KEGG) pathways [42]. Functional enrichment analysis (GO and KEGG) was also performed using ClusterProfiler. Systematic analysis of ligand‐receptor interaction was conducted using the CellPhone DB v2.0 [43].

### 2.14 Enzyme‐Linked Immunosorbent Assay (ELISA)

EC^siPiezo1^ or EC^siControl^ was co‐cultured with VSMC under the stimulation of HSS for 12 h. The EC culture medium was collected and filtered. PDGF‐BB was quantified using an ELISA assay kit (Cat No: KE10034, proteintech, USA) following the manufacturer's protocol.

### 2.15 EC Medium Concentration

The concentrated EC^siPiezo1^ and EC^siControl^ media were collected via centrifugation at 4°C for 15 min (4000 *g*/min) in an Amicon Ultra 10 KDa ultrafiltration tube after 12 h of co‐culture under HSS stimulation.

### 2.16 Western Blotting (WB) and Co‐Immunoprecipitation (Co‐IP)

Western blotting was performed following the protocol described in our previous work [30]. The primary antibodies used were GAPDH (1:5000, ab8245, Abcam, USA), β‐actin (1:2000, 66009‐I‐Ig, Proteintech, USA), H3 (1:1000, ab32356, Abcam, USA), Piezo1 (1:1000, DF12083, Affinity, USA), α‐SMA (1:1000, ab124964, Abcam, USA), SM22α (1:1000, 10493‐1‐AP, Proteintech, USA), OPN (1:2000, 22952‐1‐AP, Proteintech, USA), YAP (1:1000, 14074, Cell Signaling Technology, USA) and MMP2 (1:800, 10373‐2‐AP, Proteintech, USA). The secondary horseradish peroxidase (HRP)‐conjugated antibody (Proteintech) was diluted at 1:5000. Protein bands were quantified via densitometry using ImageJ software.

Nuclear and cytoplasmic lysates were immunoprecipitated with the specified antibody or control IgG for 2 h. Immune complexes were then collected and analyzed by standard western blotting.

### 2.17 qRT‐PCR

Total RNA was extracted from the cells and tissue samples using TRIzol reagent (Thermo, USA). cDNA was synthesized using the ABScript II cDNA First‐Strand Synthesis Kit following the manufacturer's protocol. mRNA expression levels were evaluated using SYBR Green Real‐time PCR Master Mix (TOYOBO, Osaka, Japan). Relative gene expression was normalized to that of glyceraldehyde 3‐phosphate dehydrogenase (GAPDH) or β‐actin and reported using the 2^−ΔΔCt^ method.

### 2.18 Statistical Analysis

GraphPad Prism 10 (San Diego, CA, USA) was used for statistical analysis. Data are presented as mean ± standard deviation. Quantitative variables were analyzed using the analysis of variance test among multiple groups and Student's *t*‐test between two groups. Qualitative variables were analyzed using the chi squared test. Simple linear regression analysis was performed to evaluate the association between Piezo1 expression and shear stress in aneurysms. *p* < 0.05 was considered statistically significant.

## 3 Results

### 3.1 Negative Regulation of Piezo1 by Shear Stress

To assess the relationship between shear stress and Piezo1 expression, we applied a group of shear stresses to ECs. The expression level of Piezo1 was elevated as shear stress changed from 0 dyn/cm^2^ to 30 dyn/cm^2^; however, a further increase in shear stress resulted in a decrease in Piezo1 expression (Figure 1C). This suggests that abnormally high shear stress (> 30 dyn/cm^2^) leads to downregulation of Piezo1, so we considered 30 dyn/cm^2^ shear stress as a physiological shear stress (PSS) stimulus. Consistent with findings in human IA samples, preoperative computational fluid dynamics (CFD) analysis revealed a negative correlation between aneurysmal shear stress and Piezo1 expression (Figure 1B), as abnormally high shear stress facilitates the initial formation of IA.

### 3.2 Piezo1 Ion Channels in Response to Fluid Shear Stress

Based on the characteristics of Piezo1 calcium influx, the response of Piezo1 ion channel to shear stress stimulation was verified. ECs and VSMCs were co‐cultured, and the free calcium ion concentration in ECs was measured by applying a shear stress stimulus via a flow chamber or by administering Yoda1 and GsMTx4 into the EC layer. The relative fluorescence intensity of calcium ions increased under the stimulation of 30 dyn/cm^2^ shear stress compared with the resting state (*p* < 0.001). Furthermore, Yoda1 increased the fluorescence intensity, similar to the effect of 30 dyn/cm^2^ shear stress stimulation (*p* = 0.9915). Moreover, GsMTx4 attenuated the increased intracellular calcium induced by shear stress stimulation (*p* < 0.001), consistent with the resting state (*p* = 0.9819) (Figure 1D).

Next the effect of Piezo1 knockdown on channel function was assessed by transfecting lenti‐cas9‐sgRNA into ECs. Three independent clones were analyzed by WB to check the knockdown efficiency. As shown in Figure S1, sgRNA‐11,569‐1 had the lowest expression of Piezo1, which was selected for subsequent functional analysis. In a medium supplemented with CaCl_2_, the transfected cell line was subjected to 30 dyn/cm^2^ shear stress. The Ca^2+^ fluorescence intensity decreased (*p* < 0.0001) after Piezo1 knockdown (Figure 1E).

### 3.3 EC Piezo1 Participates in VSMC Phenotype Regulation

**FIGURE 2:**
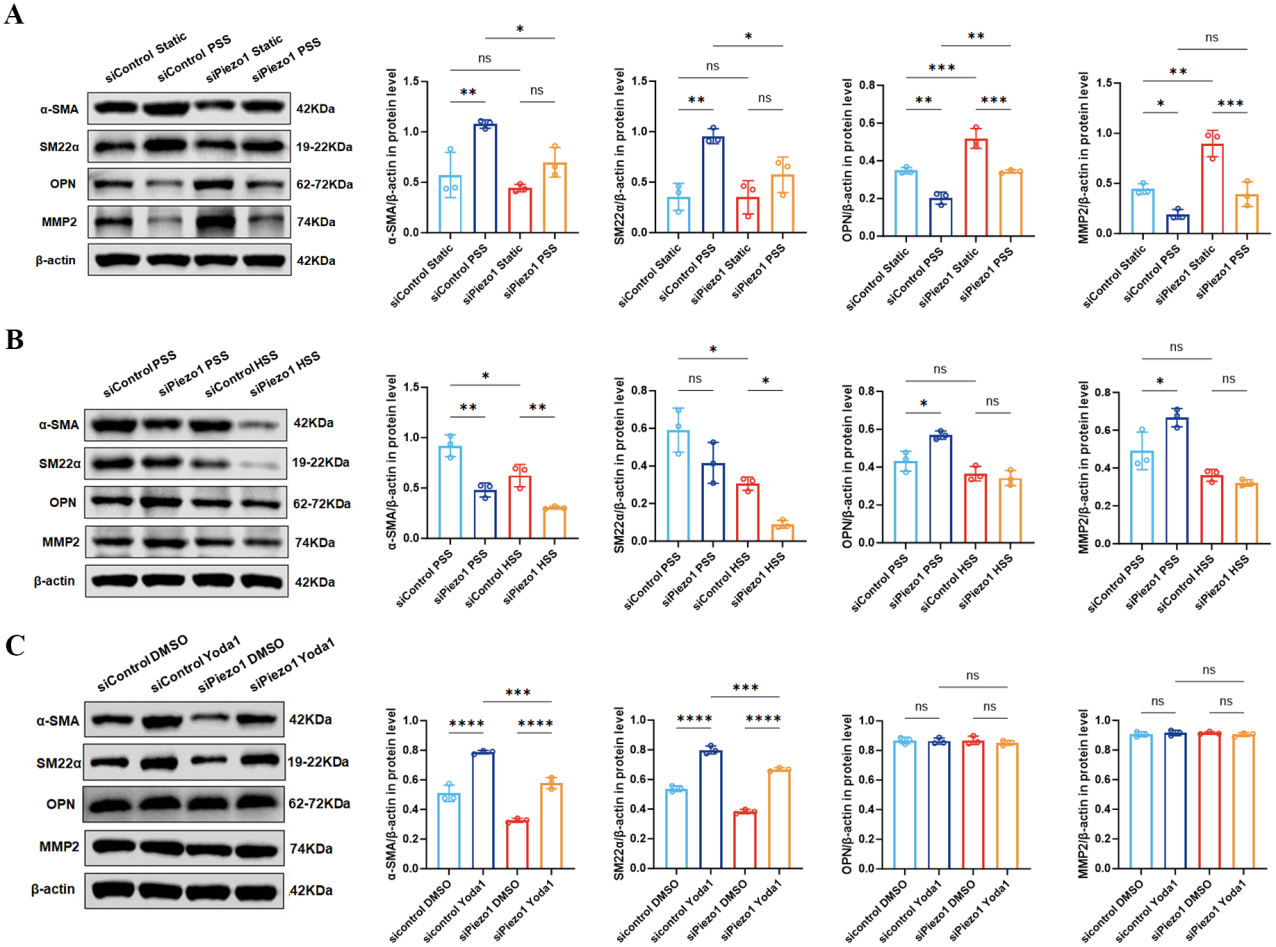
EC Piezo1 disruption promotes phenotype transformation of co‐cultured VSMCs. (A) Effects of Piezo1 knockdown on α‐SMA, SM22α, OPN, and MMP2 in VSMCs under rest and PSS conditions. (B) Effects of Piezo1 knockdown on α‐SMA, SM22α, OPN, and MMP2 in VSMCs under PSS and HSS conditions. (C) Effects of Piezo1 knockdown on α‐SMA, SM22α, OPN, and MMP2 in VSMCs under Yoda1 stimulation. *, **, ***, and **** represent *p*‐values < 0.05, 0.01, 0.001, and 0.0001 respectively.

Given that HSS stimulation decreased Piezo1 expression, the effects of EC Piezo1 on neighboring VSMCs were further investigated. The VSMC phenotype was specifically evaluated by co‐culturing ECs^siPiezo1^ and VSMCs. To exclude the influence of ECs in different states on the phenotype of co‐cultured VSMCs as previously reported [44, 45, 46], the resting state was first compared with 24‐h PSS stimulation. Expectedly, exposure of ECs^siControl^ to 30 dyn/cm^2^ shear stress (representing PSS) increased α‐SMA and SM22α expression (*p* < 0.01) and decreased OPN (*p* < 0.01) and MMP2 (*p* < 0.05) expression compared with the resting state. However, PSS did not increase the expression of α‐SMA and SM22α in VSMCs compared with the resting state when EC Piezo1 was inhibited, whereas the levels of OPN and MMP2 decreased (*p* < 0.001) (Figure 2A). Furthermore, α‐SMA and SM22α expression levels were insignificantly changed when EC Piezo1 depleted in a resting state. Moreover, the depletion of EC Piezo1 significantly downregulated α‐SMA (*p* < 0.05) and SM22α (*p* < 0.05) expression but upregulated OPN (*p* < 0.01) expression in response to PSS stimulation (Figure 2A). It also significantly increased the mRNA level but not the protein level of MMP2 (Figures 2A and S2A). These results suggest that EC Piezo1 is involved in shear stress‐mediated regulation of the VSMC phenotype, possibly by affecting contractile marker genes.

The effects of EC Piezo1 on VSMC under 12 h of HSS stimulation were further explored. WB and qRT‐PCR results showed that α‐SMA and SM22α expression were downregulated with HSS stimulation compared with that of PSS, whereas the expression of OPN and MMP2 were insignificantly changed (Figures 2B and S2B). The results suggest that HSS modulates the VSMC phenotype by reducing the expression of the contractile marker gene. Consistent with our previous findings, both α‐SMA (*p* < 0.01) and SM22α (*p* < 0.05) expression levels decreased when EC Piezo1 expression was depleted with HSS stimulation. However, OPN and MMP2 levels remained unchanged (Figures 2B and S2B). Notably, there were no significant changes, but a downward trend in both mRNA and protein levels of SM22α when EC Piezo1 was depleted after 12 h of PSS stimulation (Figures 2B and S2B), suggesting a time‐intensity dependent modulation of the VSMC phenotype by shear stress stimulation.

To eliminate the influence of other shear stress‐sensitive mechanical sensors, the Piezo1 agonist Yoda1 was used to selectively activate the Piezo1 channel. The α‐SMA (*p* < 0.0001) and SM22α (*p* < 0.0001) expression levels were upregulated when the Piezo1 channel was activated, whereas OPN expression was not affected (Figures 2C and S2C). The mRNA level of MMP2 was significantly reduced following Yoda1 treatment, but not its protein level (Figures 2C and S2C). More importantly, EC Piezo1 depletion under Yoda1 treatment significantly reduced both the mRNA and protein levels of α‐SMA and SM22α (Figures 2C and S2C), consistent with our previous results. However, the OPN and MMP2 protein levels remained unchanged (Figure 2C). This result indicates that the expression of synthetic genes may be influenced by other shear stress‐sensitive mechanoreceptors. All the above results suggest that EC Piezo1 markedly contribute to the modulation of neighboring VSMC phenotypes.

### 3.4 EC Piezo1 Knockout Promotes IA Formation in Mice

**FIGURE 3:**
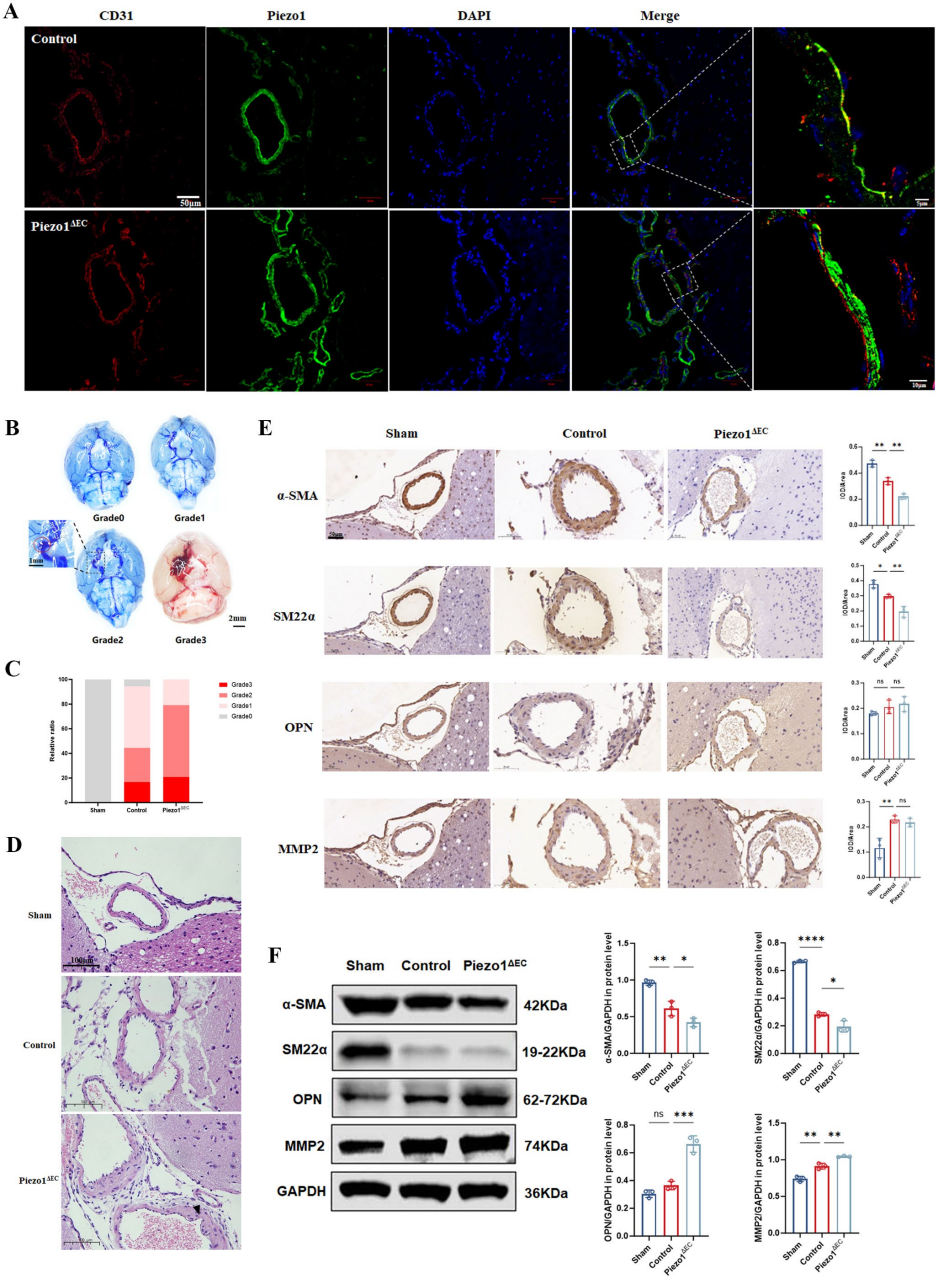
Conditional EC Piezo1 knockout promotes VSMC phenotypic transformation and IA formation in the IA mouse model. (A) Generation of Piezo1^ΔEC^ mice validated by the circle of Willis immunofluorescence. (B, C) Grading of aneurysm‐like severity of the Willis circles in sham, control, and Piezo1^ΔEC^ mice respectively. (D) HE staining of the circle of Willis in sham, control, and Piezo1^ΔEC^ mice, respectively. Black triangle represents a tendency for local lumen protrusion. (E) Immunohistochemistry of α‐SMA, SM22α, OPN, and MMP2 in the circle of Willis in sham, control, and Piezo1^ΔEC^ mice, respectively. *n* = 3 mice in each group. (F) WB of α‐SMA, SM22α, OPN, and MMP2 in the circle of Willis in sham, control, and Piezo1^ΔEC^ mice, respectively. Each sample consisted of a pool of two mice. *n* = 6 mice in each group. *, **, ***, and **** represent *p*‐values < 0.05, 0.01, 0.001, and 0.0001 respectively.

VSMC phenotypic transformation is closely related to the pathological process of IA. Therefore, we tested the role of EC Piezo1 in vivo. EC Piezo1 knockout in the circle of Willis was verified via immunofluorescence four weeks after AAV infection (Figure 3A). Three weeks after stereotactic brain injection, light microscopy revealed that the Piezo1^ΔEC^ mice had a higher aneurysmal vessel grading than the mice in the control group (Figure 3B,C). Moreover, the incidence of aneurysm in the Piezo1^ΔEC^ group (*n* = 19) was significantly higher than that in the control group (*n* = 8) (79.17% vs. 44.44%, *p* < 0.05). The arteries of the circle of Willis were harvested and sectioned for HE staining. A distinct IEL was noted in the sham group, whereas the IEL disappeared in the control and Piezo1^ΔEC^ groups. Furthermore, the middle layer of the artery was also disordered and thickened in the two groups, suggesting a synthetic phenotype of VSMC in the early stages of aneurysm formation. More importantly, compared with the control group, the vessels in the Piezo1^ΔEC^ group had irregular lumen shapes, uneven wall thickness and a tendency for local lumen protrusion. (Figure 3D). These results suggest that the absence of EC Piezo1 promotes aneurysmal vascular remodeling in mice IA models.

Next, the phenotypic markers of VSMCs in the circle of Willis were investigated. Immunohistochemistry demonstrated that α‐SMA (*p* < 0.01) and SM22α (*p* < 0.01) levels were decreased in the Piezo1^ΔEC^ group (Figure 3E). This result was confirmed using WB assays (Figure 3F). Furthermore, OPN expression levels showed an upward trend in the Piezo1^ΔEC^ group using immunohistochemistry assay but a significant increase using WB assays. (Figure 3E,F). Overall, EC Piezo1 depletion significantly promoted VSMC dedifferentiation in vivo and, consequently, IA formation in mice.

### 3.5 Differentially Expressed Receptor‐Ligand Pair Between ECs and VSMCs After EC Piezo1 Knockdown

**FIGURE 4:**
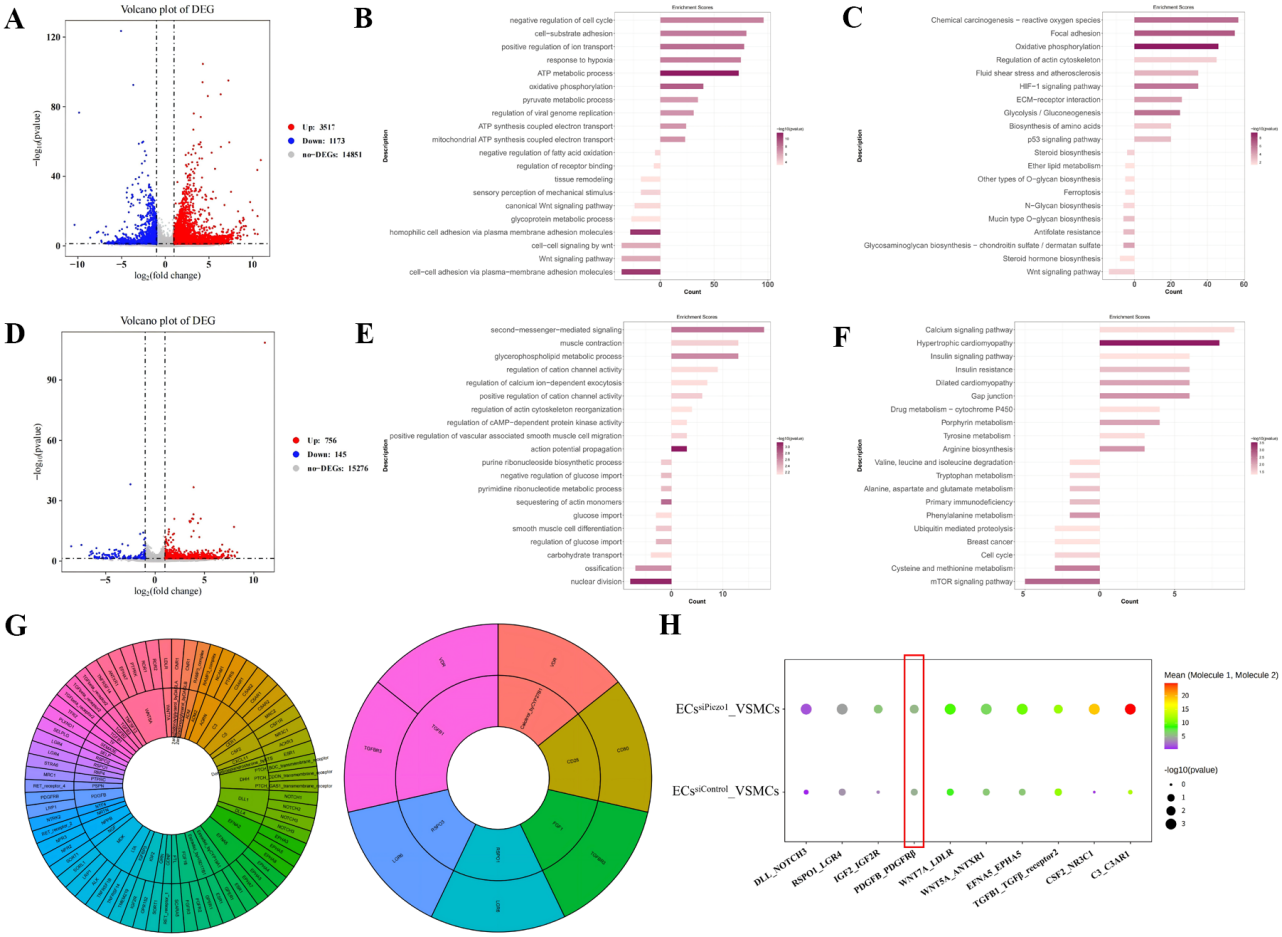
Analysis of differential gene expression and intercellular communication following EC Piezo1 knockdown. (A) Volcano plot of the differential genes in the ECs after Piezo1 knockout. (B) GO biological process analysis of DEGs in ECs. (C) KEEG enrichment analysis of DEGs in ECs. (D) Volcano plot of the differential genes in the VSMCs after EC Piezo1 knockout. (E) GO biological process analysis of DEGs in VSMCs. (F) KEEG enrichment analysis of DEGs in VSMCs. (G) The up‐ (left) and downregulated (right) receptor‐ligand pairs between ECs and VSMCs. The inner circle represents the EC ligand, whereas the outer circle represents the VSMC receptor. (H) Bubble plot of partially upregulated receptor‐ligand pairs.

Paracrine is the most common way for VSMC phenotype regulation. The previous results suggested that receptor‐ligand pair between ECs and VSMCs might participate in EC Piezo1‐induced VSMC phenotypic regulation. The flow chamber was used to apply HSS to ECs co‐cultured with VSMCs for 12 h, thereby ruling out the interference of a complex cellular environment. Subsequently, single‐cell RNA sequencing was conducted to identify changes in the receptor‐ligand pair expression after EC Piezo1 knockdown. In total, 4690 differentially expressed genes (DEGs) were identified with 3571 genes upregulated and 1173 genes downregulated in ECs, and 901 DEGs in VSMCs with 756 upregulated and 145 downregulated (Figure 4A,B). To explore the biological process of Piezo1, GO enrichment analysis and KEGG pathway analysis were performed. The biological process of GO analysis showed that EC DEGs were significantly associated with ATP metabolism, oxidative phosphorylation, cell adhesion, and positive regulation of ion transport (Figure 4C). While genes associated with glucose metabolism, VSMC differentiation, migration, and regulation of actin cytoskeleton reorganization were significantly enriched in the DEGs of VSMCs (Figure 4D). Regarding KEGG analysis, DEGs were strongly enriched in adhesive plaques, fluid shear stress, and atherosclerosis in ECs. While in VSMCs, they were enriched in calcium signaling pathways, and gap junctions (Figure 4E,F). These results indicated that EC Piezo1 depletion significantly altered the shear stress sensing of ECs, as well as VSMCs metabolism and phenotype.

To further investigate the factors responsible for the phenotypic transformation of VSMCs, an intercellular communication network between ECs and VSMCs was established, focusing on EC ligands and VSMC receptors. After EC Piezo1 depletion, 77 receptor‐ligand pairs were significantly differentially expressed, with 70 pairs upregulated and 7 downregulated (Figure 4G). Among these receptor‐ligand pairs, platelet‐derived growth factor B (PDGFB), transforming growth factor‐beta (TGFβ), notch signaling, and Wnt signaling were identified as VSMC phenotype regulators. The expression and significance level were presented as bubble plot (Figure 4H). These results suggest that the VSMC phenotypic transformation is most likely mediated by the EC‐VSMC receptor‐ligand pair.

### 3.6 PDGFB_PDGFRβ Is Involved in HSS‐Induced VSMC Phenotype Transformation and IA Progression

**FIGURE 5:**
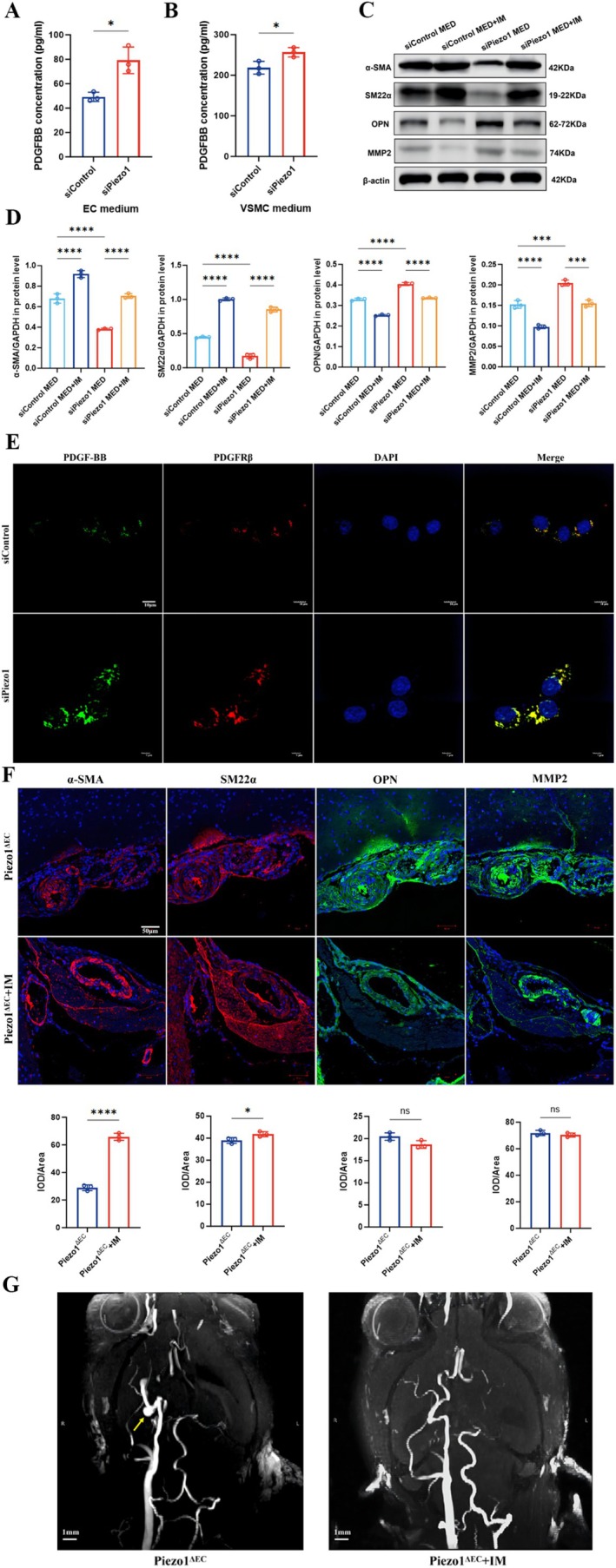
Disruption of EC Piezo1 promotes VSMC phenotype transformation and IA progression via PDGFB_PDGFRβ. The concentration of PDGF‐BB in the medium of ECs (A) and VSMCs (B) after EC Piezo1 knockdown. (C, D) The PDGFRβ antagonist imatinib mesylate reverses the effect of ECs^siPiezo1^ medium (MED) on α‐SMA, SM22α, OPN, and MMP2. (E) Immunofluorescence staining of PDGF‐BB and PDGFRβ in VSMCs after treatment with MED of ECs^siControl^ and ECs^siPiezo1^. (F) Immunofluorescence of α‐SMA, SM22α, OPN, and MMP2 in the circle of Willis of Piezo1^ΔEC^ mice after intraperitoneal injection of imatinib. *n* = 3 mice in each group. (G) 9.0 T magnetic resonance angiography shows the circle of Willis of Piezo1^ΔEC^ mice after intraperitoneal injection of imatinib (Yellow arrow represents IA). *, ***, and **** represent *p*‐values < 0.05, 0.001, and 0.0001 respectively.

Based on the forementioned findings, the receptor‐ligand pair including PDGFB_PDGFRβ, TGFβ1_TGFβRII, and DLL1/4_NOTCH3 that may lead to VSMC phenotype transformation was explored. Further analysis of ligand expression in ECs revealed that PDGFB was increased by 8.65 fold (*p* = 0.019), whereas TGFβ1 (FC = 0.731, *p* = 0.181) and DLL4 (FC = 2.9, *p* = 0.41) showed no significant upregulation. The DEGs of the above ligands were showed in Table S4. PDGFB_PDGFRβ was identified for further study for the following reasons: first, PDGFB expression was markedly increased after EC Piezo1 depletion; second, the abundance of receptors and ligands of the PDGF signaling pathway varied considerably between EC and VSMC, which was essential for determining the signaling direction. The amount of PDGF‐BB was determined using ELISA. The results showed that the PDGF‐BB concentration was elevated in both the EC (49.188 ± 3.760 pg/mL vs. 79.170 ± 10.855 pg/mL, *p* < 0 0.05) and VSMC (218.689 ± 15.796 pg/mL vs. 256.787 ± 11.661 pg/mL, *p* < 0.05) culture medium following EC Piezo1 knockdown after 12 h of HSS stimulation (Figure 5A,B). The EC medium was further concentrated and added to the VSMC cultured alone under static condition, decreasing α‐SMA (*p* < 0.0001) and SM22α (*p* < 0.0001) levels and increasing OPN (*p* < 0.0001) and MMP2 (*p* < 0.001) levels (Figure 5C,D). Subsequently, PDGF‐BB and PDGFRβ in VSMCs were labeled with green and red fluorescence, respectively. The fluorescence analysis revealed a marked increase in PDGF‐BB/PDGFRβ interaction following EC Piezo1 knockdown (Figure 5E). To elucidate the role of PDGFB_PDGFRβ ligand‐receptor pairs in this process, VSMCs were pre‐treated with 5 μM of imatinib mesylate for 2 h. Interestingly, imatinib mesylate effectively reversed the EC^siPiezo1^ medium‐induced VSMC phenotype transformation (Figure 5C,D). This result was also demonstrated in an in vivo IA model. However, the difference is that only α‐SMA (*p* < 0.0001) and SM22α (*p* < 0.05) expression levels was significantly increased (Figure 5F), suggesting that more complex mechanisms existed in the in vivo environment. More importantly, magnetic resonance angiography showed that imatinib attenuated the progression of aneurysm‐like vessels in the circle of Willis in Piezo1^ΔEC^ mice (Figure 5G). These data indicated the PDGFB_PDGFRβ ligand‐receptor pairs participate in EC Piezo1 depletion‐induced VSMC phenotype modulation and IA formation.

### 3.7 Piezo1 Promotes PDGFB Expression via the YAP/β‐Catenin Pathway

**FIGURE 6:**
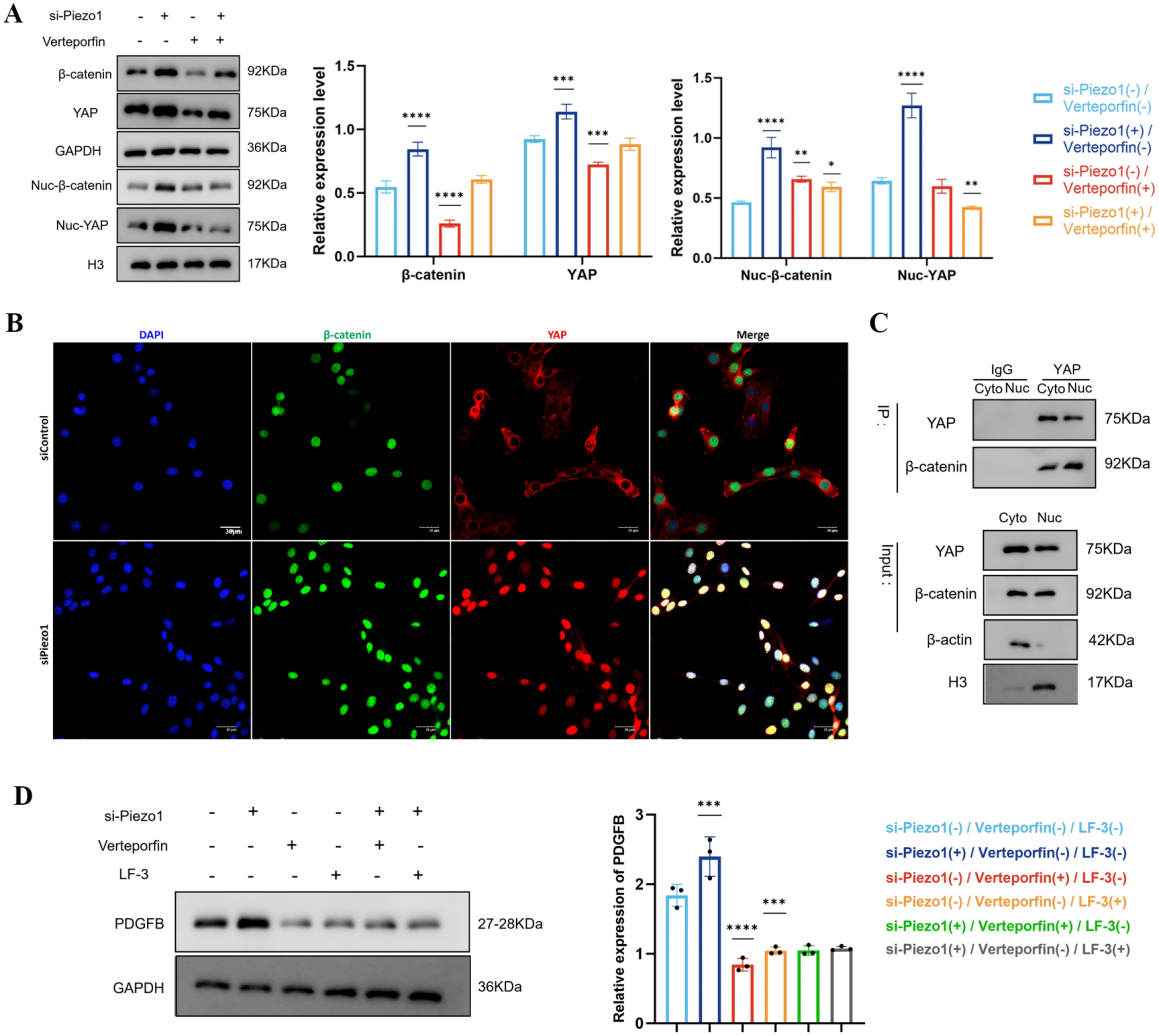
Piezo1 promotes PDGFB expression via the YAP/β‐catenin pathway. (A) Western blotting analysis of YAP and β‐catenin proteins in the cytoplasm and nucleus of ECs. Cells with Piezo1 depletion were stimulated with verteporfin for 24 h. Cyto, cytoplasm; Nuc, nucleus. β‐actin and H3 are the loading controls. (B) Immunofluorescence staining of YAP and β‐catenin in ECs after Piezo1 knockdown, as determined by confocal laser scanning microscopy. (C) Co‐immunoprecipitation analysis. Nuclear and cytoplasmic of ECs were immunoprecipitated with anti‐YAP or non‐specific IgG after Piezo1 knockdown. (D) Western blotting analysis of PDGFB proteins in the ECs. Cells were stimulated with Piezo1 inhibition, verteporfin and LF‐3. *, **, ***, and **** represent *p*‐values < 0.05, 0.01, 0.001, and 0.0001 respectively.

In order to elucidate the underlying mechanisms contributing to the increased secretion of PDGFB, we conducted an analysis of differential gene expression in endothelial cells (ECs) following the knockdown of Piezo1. Our analysis identified a total of 4690 differentially expressed genes, of which 3517 were upregulated, including YAP, Wnt9A, Wnt5A, and Wnt11, while 1173 genes were downregulated. The Wnt/β‐catenin signaling pathway is known to directly regulate PDGFB expression [47]. Additionally, YAP plays a significant role in Piezo1‐mediated mechanotransduction [48, 49]. Consequently, we investigated the expression of key factors within these signaling pathways post‐Piezo1 knockdown. Notably, the knockdown of Piezo1 resulted in a concurrent increase in YAP and β‐catenin expression, along with a significantly enhanced nuclear co‐localization of YAP and β‐catenin compared to control samples (Figure 6A,B). Furthermore, co‐immunoprecipitation (Co‐IP) assays revealed that YAP physically interacts with β‐catenin in both the nucleus and cytoplasm (Figure 6C), indicating the formation of a YAP/β‐catenin complex. Additionally, treatment with the YAP inhibitor verteporfin partially mitigated the upregulation and nuclear translocation induced by Piezo1 knockdown.

To further clarify the signaling pathway involved, verteporfin and the β‐catenin inhibitor LF‐3 were employed to inhibit signal transduction in endothelial cells (ECs) following Piezo1 knockdown. As demonstrated in Figure 6D, the increase in PDGFB expression induced by Piezo1 knockdown was effectively suppressed by both verteporfin and LF‐3. These findings collectively suggest that Piezo1 knockdown activates the YAP/β‐catenin signaling pathway in ECs, resulting in the upregulation of PDGFB expression and subsequently contributing to the formation of IAs.

Overall, we propose that HSS induces downregulation of Piezo1, which promotes PDGF‐BB secretion via the YAP/β‐Catenin signaling pathway. This leads to phenotypic transformation in neighboring VSMCs and ultimately contributes to IA formation.

## 4 Discussion

This study focused on the mechanical and biological coupling between Piezo1 and hemodynamic‐induced IA formation. The negative correlation between shear stress and Piezo1 expression was first identified. We then confirmed that Piezo1 induces Ca^2+^ influx in response to shear stress stimulation. Flow cytometry results showed that inhibition of Piezo1 gene expression decreased endoflow Ca^2+^. It was demonstrated for the first time that EC Piezo1 depletion resulted in VSMC phenotypic transformation in a co‐culture flow system. We further developed conditional EC Piezo1 knock‐out mice to validate the role of EC Piezo1 in the IA model. The morphology of the circle of Willis and the HE staining assay indicated that EC Piezo1 depletion contributed to IA progression. PDGFB_PDGFRβ ligand‐receptor pairs were then screened and identified as being involved in the phenotypic transformation of VSMCs via intercellular communication analysis. Finally, the possible mechanism of PDGF‐BB overexpression were explored. Based on these findings, we propose a novel mechanism in which HSS induces Piezo1 downregulation, thereby enhancing PDGF‐BB expression via activation of the YAP/β‐catenin signaling pathway. Then the PDGF‐BB is subsequently released in a paracrine manner, which leads to phenotypic transformation in neighboring VSMCs and ultimately contributes to IA formation.

It is evidenced that abnormal hemodynamics is closely related to the occurrence of IA. Consistent with the observation of de novo IA following unilateral carotid artery ligation [50, 51], researchers have successfully constructed IA in an animal model by ligating the unilateral carotid artery, which confirmed the hemodynamic hypothesis of IA formation [52, 53]. Hemodynamic studies have been further quantified with the application of CFD analysis to the medical field. One of the most attractive parameters was wall shear stress, a frictional force due to the viscosity of blood flow in the endothelium. Studies on the correlation between IA location and shear stress intensity have suggested that HSS provided critical conditions for de novo IA formation and triggered IEL rupture, the earliest pathological change in IA formation [14, 54, 55]. Although it is unclear how HSS initiates IA, the HSS‐induced phenotypic transformation of VSMCs may provide some clues, as VSMC dedifferentiation is considered the major mechanism of pathological vascular remodeling in IA [56].

VSMCs develop and differentiate in response to shear stress stimulation until they can maintain vasodilatory and constrictive properties to regulate blood flow and pressure. It was confirmed via in vitro experiments that the application of PSS to ECs promoted the differentiation of co‐cultured VSMCs from synthetic to contractile phenotypes. In the resting state, however, ECs decreased in the expression of VSMC contractile markers [45, 46]. Surprisingly, a similar result was also found with HSS stimulation [57]. Our results are in line with this viewpoint. These findings suggest that the regulation of the VSMC phenotype by ECs mainly depends on the shear stress intensity. We also found that the decrease in SM22α expression required a longer intervention or higher intensity of shear stress stimulation compared with α‐SMA, indicating the time‐ and intensity‐dependent character of phenotypic markers. This view was also supported by OPN and MMP2 expression, as there was only a slight decreasing trend when ECs were exposed to a shear stress of 60 dyn/cm^2^. Yuya et al. [57] reported a significant decrease in MMP2 expression when the shear stress was further increased to 200 dyn/cm^2^. However, this result did not indicate that HSS inhibited inflammation because the MMP2/TIMP‐2 ratio remained unchanged, as previously reported. These results indicate that the absence of contractile properties is an early manifestation of HSS‐induced VSMC phenotypic transformation.

Piezo1, a novel ion channel, is inherently mechanosensitive. It is widely distributed in the cell membrane of the vascular wall, and EC Piezo1 directly senses shear stress stimulation. Consistent with previous studies, we observed a shear stress‐mediated activation of Piezo1, which consequently led to calcium influx. Expectedly, calcium influx was also observed following the stimulation of the Piezo1 agonist Yoda1, whereas the inhibitor GsMTx4 prevented shear stress‐induced calcium ion influx. Additionally, the inhibition of Piezo1 gene expression had similar effects to the Piezo1 channel inhibitor GsMTx4. However, GsMTx4 was more effective at preventing calcium ion influx. Therefore, we have to emphasize the accuracy of inhibiting Piezo1 gene expression rather than the non‐specific inhibitor GsMTx4, which was important to our research. More importantly, the shear stress‐mediated regulation of Piezo1 in transcriptional levels was confirmed in the present study. This can be understood by the fact that shear stress intensity regulates intracellular calcium levels and thus intracellular biological functions through Piezo1 expression. Disruption of calcium ion homeostasis may explain why both abnormally high and low shear stress levels are believed to promote IA progression.

Currently, there is no direct evidence that EC Piezo1 regulates the VSMC phenotype. However, previous studies have suggested that EC Piezo1 is likely involved in this process. Caolo et al. [58] found that EC Piezo1 deficiency changed the notch signaling pathway, which is closely related to vascular development and homeostatic regulation. Similar results have also been reported in in vivo studies. Wang et al. [21] showed that low osmolarity‐mediated stretching of EC membranes upregulated Jagged1 expression via the Piezo1/Ca^2+^/AKT/mTOR signaling pathway in the pulmonary artery. Our research revealed that inhibiting the gene expression of EC Piezo1 resulted in the de‐differentiation of the VSMCs, regardless of stimulation by PSS, HSS, or Yoda1. Noteworthy, the mechanisms underlying the phenotypic transformation may differ under these conditions. Specifically, the decreased contractile marker protein levels contributed entirely to the phenotypic change under HSS stimulation. For PSS stimulation, however, increased expression of OPN was also involved. According to these findings, we speculate that the level of Ca^2+^ influx is too low to initiate OPN gene regulation, because HSS further inhibits Piezo1 expression. We also found an increase in the inflammatory marker MMP2 in the IA mouse model, which was not observed in the in vitro experiments. Therefore, we speculated that this may be related to the following reasons: first, the shear stress varies with the cardiac cycle in vivo rather than a constant HSS environment; second, to accelerate IA formation, elastase injection was used to weaken the vessel wall, causing further inflammatory responses. Consistent with the VSMC dedifferentiation, mice in the Piezo1^ΔEC^ group had worse pathology of the circle of Willis and a higher incidence of IA. Interestingly, our study appears to contradict a recent study by Qian et al. [59]. They found that the Piezo1 inhibitor GsMTx4 could reduce the incidence of abdominal aortic aneurysm due to decreased activity of Piezo1 in VSMCs by inhibiting the mechanically solid‐like state of VSMCs. It is far from the mechanisms of IA. Additionally, the effect of GsMTx4 is more complex than the cell‐selective depletion of Piezo1.

Intracellular communication between ECs and VSMCs is crucial for maintaining vascular homeostasis and exerting physiological functions, whereas aberrant stimulation can alter this communication and lead to vascular dysfunction [60]. Previous studies have shown that the PDGFB of ECs can be upregulated by both high and low shear stress [61, 62]. In this study, we found that the depletion of EC Piezo1 also upregulated PDGFB. Based on the negative regulation of Piezo1 expression by the shear stress, we propose that EC Piezo1 is involved in PDGFB regulation in a shear stress‐dependent manner. We also found that elevated PDGF‐BB concentration promoted VSMC phenotypic transformation through PDGFRβ while competitive inhibition of PDGFRβ alleviated the severity of IA in Piezo1^ΔEC^ mice. Several studies also support our findings. It has been demonstrated that PDGF‐BB inhibits the expression of VSMC contraction marker genes at the transcriptional level through multiple complementary inhibition pathways, including Kruppel‐like factor 4 (KLF4), HERP1, and ELK1 phosphorylation [63, 64, 65]. More importantly, Mielko et al. [66] found that PDGF‐BB was expressed in ECs with HSS loaded in the neck of the IA, which was correlated closely with the dedifferentiation of VSMCs at this site. This study further supports the involvement of PDGFB in HSS‐induced phenotypic transformation of VSMCs during IA pathogenesis.

As a principal transcriptional co‐activator in the Hippo signaling cascade, YAP serves as a central regulatory node in diverse physiological and pathological processes. The interaction between Piezo1 and YAP has emerged as a focal point in contemporary mechanotransduction studies. Accumulating evidence indicates that Piezo1‐mediated calcium influx initiates YAP activation through both direct ionic signaling and calcium‐dependent enzymatic cascades [67, 68]. Moreover, a recent study indicated that Piezo1 senses changes in environmental mechanical forces and regulates YAP distribution through the microfilament cytoskeleton network, as demonstrated by Duan et al. [49]. He found that activation of Piezo1 in tumor cells led to nuclear accumulation of YAP. However, prolonged activation resulted in YAP nuclear export and microfilament depolymerization. Our experimental data suggest that inhibition of Piezo1 enhances YAP nuclear translocation, which seems to support the above viewpoint from an opposite perspective. Mechanistically, we identified concurrent upregulation and nuclear co‐localization of β‐catenin with activated YAP, which aligns with observations by Viola et al. [69] in melanoma. This transcriptional complex formation assumes particular significance given established evidence that transcriptional activity of β‐catenin directly regulates endothelial expression of PDGFB [47]. This finding demonstrates that Piezo1 inhibition‐mediated upregulation of PDGFB is mechanistically dependent on the YAP/β‐catenin signaling axis.

Elucidating the biomechanical coupling of HSS‐induced IA formation represents a significant advance with promising implications for therapeutic intervention. First, by revealing the negative regulation of HSS by Piezo1 and the subsequent promotion of VSMC phenotypic transformation, the theoretical basis of aneurysmal hemodynamics was refined. More importantly, by identifying PDGFB_PDGFRβ as a key communicator that modulates VSMC phenotypes in the absence of EC Piezo1, our findings provide a novel molecular target for potential therapeutic strategies. Targeting PDGFB_PDGFRβ may lead to the development of innovative approaches to modify the behavior of VSMCs, which are crucial in IA pathogenesis. Therapies designed to downregulate PDGFB expression in ECs can inhibit VSMC dedifferentiation and prevent aneurysm formation. Similarly, interventions for disrupting the PDGFRβ of VSMCs could restore the contractile phenotype to suppress collagen secretion.

## 5 Conclusions

In summary, we found that HSS downregulated EC Piezo1 expression, thereby inducing phenotypic transformation of VSMCs and contributing to the IA formation. Mechanistically, we demonstrated that the inhibition of EC Piezo1 gene expression increased PDGF‐BB levels via YAP/β‐catenin pathway, which in turn promoted VSMC dedifferentiation by binding to PDGFRβ.

## Author Contributions

Z.L., S.L., X.Y., and H.T. carried out experimental research; Q.H., F.X., and C.W. collected samples of patients. Data were analyzed by Z.L. and S.Z. Z.L. and S.L. drafted the manuscript; Q.H. and F.X. conceived and designed the research; Q.H. initiated and organized this study.

## Funding

This study was supported by the National Natural Science Foundation of China (No. 82371314 & No. 82071279).

## Ethics Statement

This study was performed in line with the principles of the Declaration of Helsinki. Approval was granted by the Ethics Committee of Naval Medical Center. (No. 2025Q050).

## Consent

Informed consent was obtained from all individual participants included in the study. The authors affirm that human research participants provided informed consent for publication.

## Conflicts of Interest

The authors declare no conflicts of interest.

## Supporting information


**Figure S1:** The efficiency of Piezo1 knockdown in Lenti‐Cas9‐sgRNA‐transfected ECs is determined by WB assay.

**Figure S2:** Disruption of EC Piezo1 promotes changes in phenotypic marker genes at the mRNA level in co‐cultured VSMCs.
(A) Effects of Piezo1 knockdown on α‐SMA, SM22α, OPN, and MMP2 gene expression in VSMCs under rest and PSS conditions. (B) Effects of Piezo1 knockdown on α‐SMA, SM22α, OPN, and MMP2 gene expression in VSMCs under PSS and HSS conditions. (C) Effects of Piezo1 knockdown on α‐SMA, SM22α, OPN, and MMP2 gene expression in VSMCs under Yoda1 stimulation. *, **, ***, and **** represent *p*‐values < 0.05, 0.01, 0.001 and 0.0001 respectively.

**Table S1:** The basic information and clinical characteristics of IA patients.

**Table S2:** The hemodynamic parameters and Piezo1 expression of IA sample.

**Table S3:** Sequences of sgRNAs for Piezo1.

**Table S4:** DEGs of ligands associated with VSMC phenotypic regulation after EC Piezo1 knockout.

## Data Availability

The datasets generated during the current study are not publicly available due to copyright protection but are available from the corresponding author upon reasonable request.
