# Supplementary figures and images for: High Shear Stress‐Induced Endothelial Piezo1 Downregulation Promotes Intracranial Aneurysm Formation via the PDGF‐BB/PDGFRβ Paracrine Signaling Pathway
Source: CNS Neurosci Ther. 2025 Dec 28;31(12):e70715. doi: 10.1002/cns.70715 (PMC12745340; doi:10.1002/cns.70715)

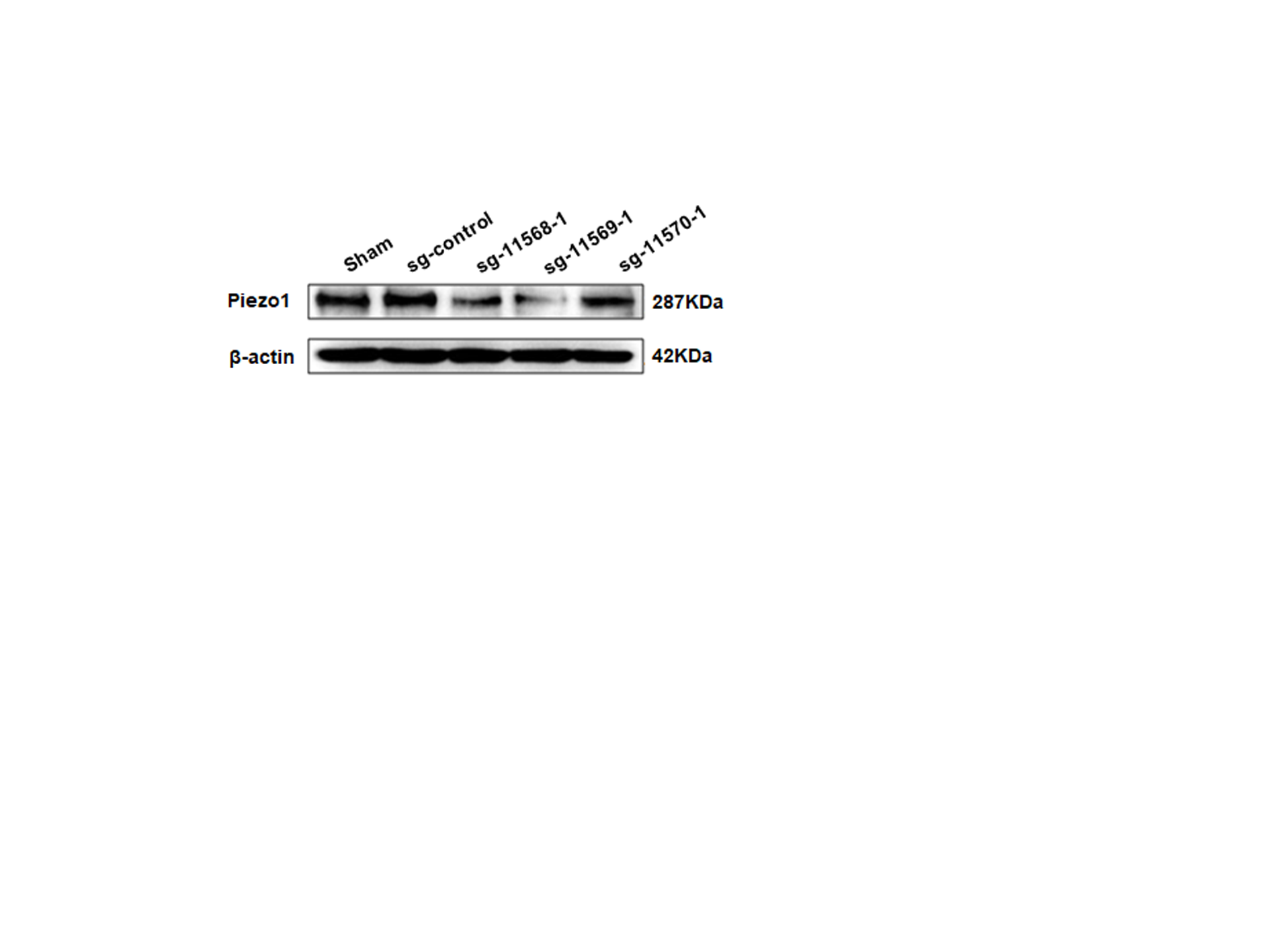

Supplement: Supplementary file 1 — Figure S1: The efficiency of Piezo1 knockdown in Lenti‐Cas9‐sgRNA‐transfected ECs is determined by WB assay. [file CNS-31-e70715-s006.tif]

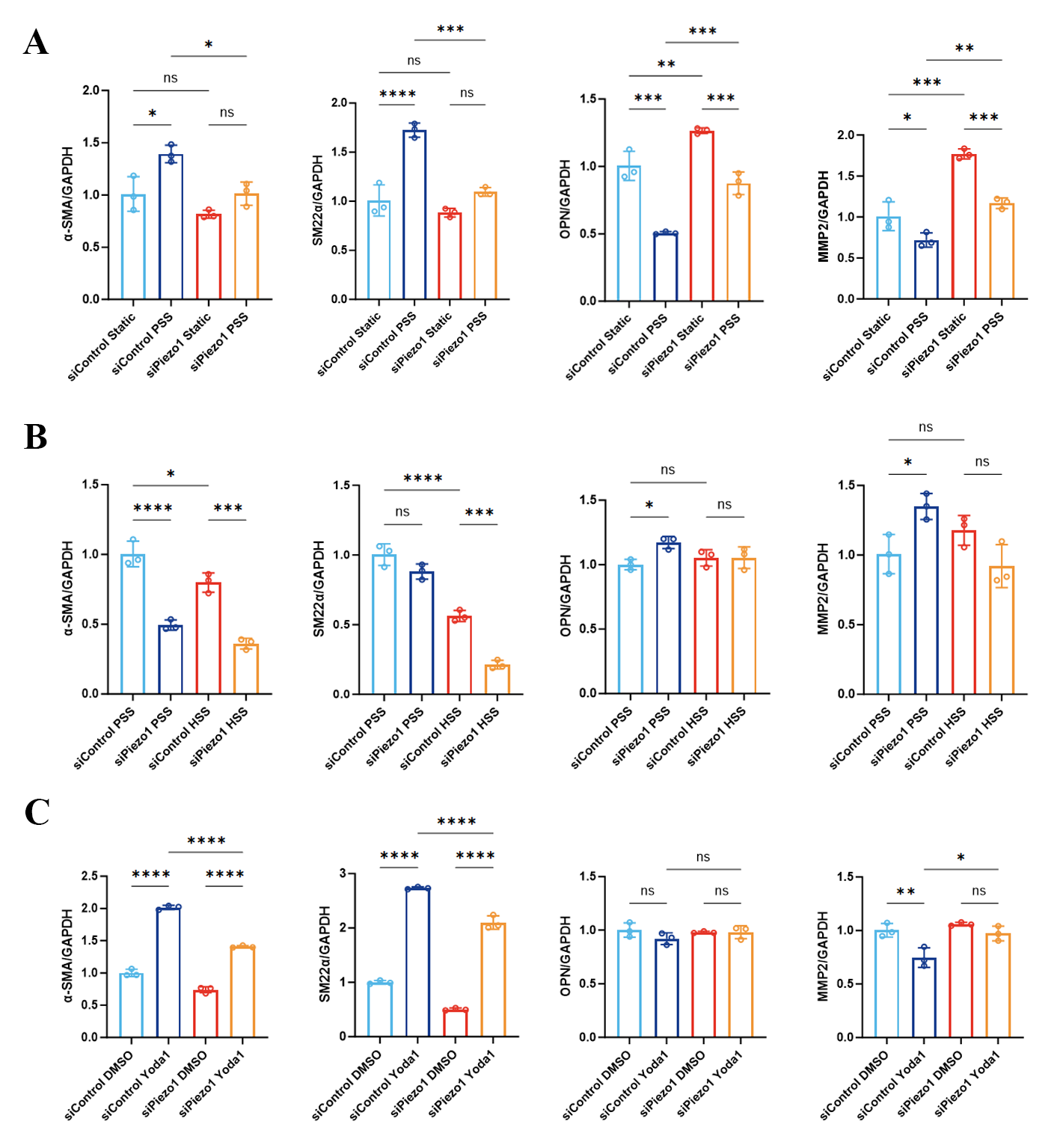

Supplement: Supplementary file 2 — Figure S2: Disruption of EC Piezo1 promotes changes in phenotypic marker genes at the mRNA level in co‐cultured VSMCs. (A) Effects of Piezo1 knockdown on α‐SMA, SM22α, OPN, and MMP2 gene expression in VSMCs under rest and PSS conditions. (B) Effects of Piezo1 knockdown on α‐SMA, SM22α, OPN, and MMP2 gene expression in VSMCs under PSS and HSS conditions. (C) Effects of Piezo1 knockdown on α‐SMA, SM22α, OPN, and MMP2 gene expression in VSMCs under Yoda1 stimulation. *, **, ***, and **** represent p‐values < 0.05, 0.01, 0.001 and 0.0001 respectively. [file CNS-31-e70715-s004.tif]
