# Supplementary material for: High Shear Stress‐Induced Endothelial Piezo1 Downregulation Promotes Intracranial Aneurysm Formation via the PDGF‐BB/PDGFRβ Paracrine Signaling Pathway
Source: CNS Neurosci Ther. 2025 Dec 28;31(12):e70715. doi: 10.1002/cns.70715 (PMC12745340; doi:10.1002/cns.70715)
Supplement: Supplementary file 3 — Table S1: The basic information and clinical characteristics of IA patients. [file CNS-31-e70715-s005.docx]

Supplemental Table1 The basic information and clinical characteristics of IA patients

| **ID** | **Gender/Age** | **Smoking** | **HBP** | **Location** | **Size (mm)** |
| --- | --- | --- | --- | --- | --- |
| R1 | M, 59 | Yes | No | AComA | 10 |
| R2 | M, 38 | Yes | No | MCA | 19 |
| R3 | F, 61 | No | No | AComA | 10 |
| R4 | M, 54 | No | No | MCA | 15 |
| R5 | F, 62 | No | Yes | MCA | 10 |
| R6 | M, 61 | No | No | AComA | 6 |
| R7 | M, 66 | No | Yes | MCA | 6 |
| U1 | F, 61 | No | Yes | MCA | 20 |
| U2 | F, 54 | No | Yes | MCA | 18 |
| U3 | M, 45 | Yes | No | ACA | 23 |
| U4 | F, 55 | No | Yes | PComA | 7 |
| U5 | F, 75 | No | Yes | AComA | 6 |
| U6 | F, 60 | No | No | PComA | 7 |

Abbreviations: R, ruptured aneurysm; U, unruptured aneurysm; F, female; M, male; AcomA, anterior communicating artery; MCA, middle cerebral artery; ACA, anterior cerebral artery; PComA, posterior communicating artery.
