# Supplementary material for: High Shear Stress‐Induced Endothelial Piezo1 Downregulation Promotes Intracranial Aneurysm Formation via the PDGF‐BB/PDGFRβ Paracrine Signaling Pathway
Source: CNS Neurosci Ther. 2025 Dec 28;31(12):e70715. doi: 10.1002/cns.70715 (PMC12745340; doi:10.1002/cns.70715)
Supplement: Supplementary file 4 — Table S2: The hemodynamic parameters and Piezo1 expression of IA sample. [file CNS-31-e70715-s001.docx]

Supplemental Table2 The hemodynamic parameters and Piezo1 expression of IA sample

| **ID** | **Pressure** | **WSS**  **(AN)** | **WSS**  **(PAR)** | **NWSS**  **(AN/PAR)** | **LSA** | **OSI** | **Piezo1 FPKM** |
| --- | --- | --- | --- | --- | --- | --- | --- |
| R1 | 138.164 | 1.31578 | 7.26172 | 0.181193987 | 0.589105 | 0.048095 | 24.4292 |
| R2 | 662.241 | 0.352211 | 3.05199 | 0.11540372 | 0.98873 | 0.042241 | 25.9903 |
| R3 | 221.014 | 2.44879 | 8.56457 | 0.285920951 | 0.24674 | 0.094568 | 22.0318 |
| R4 | 410.151 | 2.40524 | 6.38989 | 0.376413365 | 0.364713 | 0.112127 | 27.5953 |
| R5 | 915.009 | 2.01743 | 5.55723 | 0.363027983 | 0.288349 | 0.113468 | 23.4666 |
| R6 | 307.088 | 2.71236 | 5.43862 | 0.498722102 | 0.051649 | 0.015742 | 16.4582 |
| R7 | 271.364 | 1.65332 | 9.00407 | 0.183619186 | 0.118282 | 0.031956 | 28.0606 |
| U1 | 625.367 | 2.692 | 5.86008 | 0.459379394 | 0.175802 | 0.067219 | 28.3936 |
| U2 | 2281.14 | 8.54304 | 36.6137 | 0.233329054 | 0.225982 | 0.037613 | 27.2263 |
| U3 | 492.83 | 0.679636 | 10.0997 | 0.067292692 | 0.962057 | 0.041798 | 26.9654 |
| U4 | 1345.47 | 6.53285 | 17.0826 | 0.382427148 | 0.127773 | 0.088508 | 22.0642 |
| U5 | 1962.12 | 5.1834 | 12.1866 | 0.425336025 | 0.066999 | 0.052552 | 14.2368 |
| U6 | 2709.12 | 19.1065 | 29.5162 | 0.647322487 | 0.000311 | 0.038487 | 15.2523 |

Abbreviations: R, ruptured aneurysm; U, unruptured aneurysm; WSS, wall shear stress; NWSS, normalized wall shear stress; AN, aneurysm; PAR, parent artery; LSA, low wall shear stress area; OSI, Oscillatory Shear Index; FPKM,
