# Supplementary material for: High Shear Stress‐Induced Endothelial Piezo1 Downregulation Promotes Intracranial Aneurysm Formation via the PDGF‐BB/PDGFRβ Paracrine Signaling Pathway
Source: CNS Neurosci Ther. 2025 Dec 28;31(12):e70715. doi: 10.1002/cns.70715 (PMC12745340; doi:10.1002/cns.70715)
Supplement: Supplementary file 5 — Table S3: Sequences of sgRNAs for Piezo1. [file CNS-31-e70715-s003.docx]

Supplemental Table3 Sequences of sgRNAs for Piezo1

| Sequence (5’-3’) | |
| --- | --- |
| sgRNA-11568-1 | CCTACACACCGTGCCTCACC |
| sgRNA-11569-1 | GGTAACATGTCCTGGATAAA |
| sgRNA-11570-1 | ACCACCAGGTGCAGATGGCC |
| sgRNA-control | CGCTTCCGCGGCCCGTTCAA |
