# Supplementary material for: High Shear Stress‐Induced Endothelial Piezo1 Downregulation Promotes Intracranial Aneurysm Formation via the PDGF‐BB/PDGFRβ Paracrine Signaling Pathway
Source: CNS Neurosci Ther. 2025 Dec 28;31(12):e70715. doi: 10.1002/cns.70715 (PMC12745340; doi:10.1002/cns.70715)
Supplement: Supplementary file 6 — Table S4: DEGs of ligands associated with VSMC phenotypic regulation after EC Piezo1 knockout. [file CNS-31-e70715-s002.docx]

**Supplemental Table4** DEGs of ligands associated with VSMC phenotypic regulation after EC Piezo1 knockout

| **GeneName** | **Chr** | **Strand** | **Start** | **End** | **FC(EC_intervention/EC_control)** | **pvalue** | **EC1-siPiezo1** | **EC2-siPiezo1** | **EC3-siPiezo1** | **EC1-NC** | **EC2-NC** | **EC3-NC** |
| --- | --- | --- | --- | --- | --- | --- | --- | --- | --- | --- | --- | --- |
| Tgfb1 | 7 | + | 25687002 | 25705077 | 0.730827056 | 0.180988 | 0.884615 | 0.523599 | 1.4074 | 0.93443 | 1.5647 | 1.36456 |
| Pdgfb | 15 | - | 79995874 | 80014977 | 8.654412499 | 0.018947 | 0.478157 | 0.107961 | 0.596971 | 0 | 0.11017 | 0 |
| Dll1 | 17 | - | 15367354 | 15376872 | 15.4947296 | 0.00063 | 0.738374 | 1.73431 | 2.72014 | 0.212727 | 0 | 0.094344 |
| Dll4 | 2 | + | 1.19E+08 | 1.19E+08 | 2.902369505 | 0.407355 | 0.213565 | 0 | 0.110794 | 0 | 0.092093 | 0 |
| Wnt7a | 6 | - | 91363981 | 91411363 | 10.56299204 | 0.00077 | 0.711956 | 0.735109 | 0.358445 | 0 | 0.143768 | 0 |
| Wnt5a | 14 | + | 28504750 | 28527448 | 2.250641717 | 7.06E-05 | 4.17405 | 3.77408 | 4.14808 | 1.24244 | 1.77734 | 2.33811 |
